# Circular RNAs in sepsis-induced acute lung injury: emerging mechanisms and therapeutic potential

**DOI:** 10.3389/fimmu.2026.1718164

**Published:** 2026-02-18

**Authors:** Qinghai Liu, Yiyan Wang, Haitang Liao, Dongsheng Ren, Wenhui Guo, Jianzhong Xu, Chenyang Duan, Zhenchun Luo, Wen Jiang

**Affiliations:** 1 Department of Critical and Emergency Medicine, Chongqing Hospital of Traditional Chinese Medicine, Chongqing, China; 2 Famous Physicians Clinic, Chongqing Hospital of Traditional Chinese Medicine, Chongqing, China; 3 Department of Anesthesiology, The Second Affiliated Hospital of Chongqing Medical University, Chongqing, China; 4 Basic Medicine Research and Innovation Center for Novel Target and Therapeutic Intervention, Ministry of Education, The Second Affiliated Hospital of Chongqing Medical University, Chongqing, China

**Keywords:** biomarkers, circular RNAs, immunological regulation, programmed cell death, sepsis-induced acute lung injury

## Abstract

Sepsis-induced acute lung injury (ALI) remains a leading cause of mortality in critically ill patients and is characterized by dysregulated inflammation, immune imbalance, and alveolar–capillary barrier dysfunction. Emerging evidence suggests that circular RNAs (circRNAs), a class of stable and highly conserved non-coding RNAs, play important regulatory roles in inflammatory diseases; however, their contributions to sepsis-associated lung injury have not yet been systematically summarized. In this review, we provide a comprehensive overview of circRNA biogenesis, classification, and regulatory properties, with a particular focus on their context-dependent functions in the septic lung. We discuss how circRNAs participate in the coordination of cell fate decisions, immune responses, and barrier integrity during sepsis, and highlight their potential as diagnostic biomarkers and therapeutic targets. Importantly, we also address current technical and translational challenges, including detection specificity, disease heterogeneity, and limited clinical validation. By integrating mechanistic insights with translational perspectives, this review aims to clarify the emerging role of circRNAs in sepsis-induced ALI and to outline key directions for future research.

## 1 Introduction

Sepsis remains a major global health burden, affecting millions of patients annually and accounting for a substantial proportion of intensive care unit admissions (1, 2). Acute lung injury (ALI), and its more severe form acute respiratory distress syndrome (ARDS), is one of the most common and devastating complications of sepsis (3, 4). Despite advances in supportive care, the morbidity and mortality of sepsis-induced ALI remain alarmingly high, with mortality rates ranging from 30% to 40% in severe cases. The lack of effective targeted therapies underscores the urgent need to elucidate the molecular mechanisms underlying this condition (5, 6). The pathophysiology of sepsis-associated ALI is highly complex and multifactorial. Hallmark features include the overwhelming release of pro-inflammatory mediators, also referred to as the “cytokine storm,” leading to widespread tissue injury (7, 8). The disruption of endothelial and epithelial barriers further exacerbates alveolar-capillary leakage, pulmonary edema, and impaired gas exchange. In addition, dysregulated immune responses, coupled with diverse forms of programmed cell death—including apoptosis, pyroptosis, and the recently recognized ferroptosis—play critical roles in disease progression (9, 10). These intertwined processes highlight the need to explore regulatory networks that orchestrate inflammation, immune responses, and cell death in the septic lung.

In recent years, non-coding RNAs (ncRNAs) have emerged as crucial regulators of gene expression in both physiological and pathological contexts, fundamentally reshaping our understanding of post-transcriptional regulation (11, 12). Among these, microRNAs (miRNAs) and long non-coding RNAs (lncRNAs) have been extensively investigated in the setting of sepsis and acute lung injury (ALI), where they influence diverse biological processes including immune cell differentiation, inflammatory cytokine production, oxidative stress responses, and apoptosis (13–15). These discoveries have underscored the centrality of ncRNA-mediated regulatory networks in determining disease severity and outcomes. Circular RNAs (circRNAs), a relatively novel subclass of endogenous ncRNAs generated through a unique back-splicing mechanism, have gained increasing attention as key players in gene regulation (16). Unlike their linear counterparts, circRNAs form covalently closed continuous loops lacking 5′ caps and 3′ poly(A) tails, which confer exceptional stability against exonuclease-mediated degradation. This structural resilience allows circRNAs to accumulate in tissues, circulate in plasma, and even be packaged into extracellular vesicles such as exosomes, thereby facilitating intercellular communication (17–19). Their abundance, evolutionary conservation, and cell-type- or tissue-specific expression patterns further highlight their functional importance. Functionally, circRNAs exert multifaceted regulatory roles. They are well recognized as competing endogenous RNAs (ceRNAs) that sponge miRNAs, thereby relieving repression of downstream target mRNAs (20, 21). Beyond this, circRNAs interact with RNA-binding proteins (RBPs), serving as molecular scaffolds or decoys to modulate RNA stability, alternative splicing, and protein localization (22). Emerging evidence also suggests that certain circRNAs participate in transcriptional regulation of their parental genes or undergo cap-independent translation through internal ribosome entry sites (IRES) or N6-methyladenosine (m6A)-mediated initiation, giving rise to biologically active peptides. These diverse mechanisms position circRNAs as versatile regulators within cellular networks (23–25). Given these unique features, circRNAs are now recognized as promising biomarkers and therapeutic targets in a wide range of pathological conditions, including cancer, cardiovascular disease, neurological disorders, and inflammatory syndromes (26–28). In the context of sepsis-induced ALI, accumulating evidence indicates that circRNAs play pivotal roles in orchestrating pathological processes such as programmed cell death (apoptosis, ferroptosis, pyroptosis), macrophage polarization, immune checkpoint regulation, and maintenance of epithelial and endothelial barrier integrity (29–31). However, despite the rapid progress in preclinical studies, this research field remains in its infancy. Comprehensive investigations are needed to integrate current mechanistic insights, validate candidate circRNAs in large patient cohorts, and explore translational opportunities that may ultimately lead to novel diagnostic tools and therapeutic strategies for this devastating condition.

This review aims to summarize the emerging roles of circRNAs in sepsis-induced acute lung injury, with a particular focus on their regulatory mechanisms, involvement in cell death and immune responses, and potential as diagnostic biomarkers or therapeutic targets. By consolidating recent advances, we seek to provide a framework for understanding how circRNA-based regulatory networks contribute to the pathogenesis of sepsis-associated ALI and to identify future directions for research and clinical translation.

## 2 Background on circRNAs

### 2.1 Biogenesis and characteristics

Circular RNAs (circRNAs) represent a unique class of endogenous non-coding RNAs that have emerged as important regulatory molecules in recent years (32). They are generated through a non-canonical splicing event known as back-splicing, in which a downstream splice donor site is covalently linked to an upstream splice acceptor site. This unconventional process gives rise to a covalently closed continuous loop lacking both the canonical 5′ cap and 3′ poly(A) tail characteristic of linear RNAs (33, 34). The circular structure not only distinguishes circRNAs structurally but also provides them with inherent resistance to exonuclease-mediated degradation, thereby conferring remarkable stability and often longer half-lives compared with their linear counterparts. Beyond their unusual structure, circRNAs exhibit several additional features that highlight their biological relevance. They are evolutionarily conserved across species, suggesting that their functions are under selective pressure and are biologically significant (35–37). CircRNAs also display spatiotemporal specificity, with distinct expression patterns depending on tissue type, developmental stage, and physiological or pathological conditions. Such expression specificity points toward finely tuned regulatory roles in diverse cellular contexts (38, 39). CircRNAs are distributed in various cellular compartments, with cytoplasmic circRNAs frequently acting as miRNA sponges or interacting with RNA-binding proteins, while nuclear circRNAs have been implicated in regulating transcription and alternative splicing. Notably, circRNAs are not restricted to intracellular compartments; they are also found in extracellular vesicles such as exosomes, as well as in peripheral body fluids including blood and saliva (40, 41). This extracellular stability enhances their potential as easily accessible, non-invasive biomarkers for disease diagnosis and prognosis. Collectively, these properties make circRNAs more than just molecular curiosities; they represent a functionally versatile and clinically relevant class of ncRNAs. Their stability, conservation, and cell-type-specific expression patterns underscore their significance as key regulatory elements in cellular homeostasis and disease, and their detectability in circulation highlights their potential utility as both mechanistic regulators and clinically actionable biomarkers.

In addition to their shared circular structure, circRNAs constitute a highly heterogeneous population that can be classified according to their genomic origin and molecular composition, which in turn dictates their subcellular localization and functional repertoire (42). Based on their biogenesis, circRNAs are generally categorized into exon-derived circRNAs (ecircRNAs), circular intronic RNAs (ciRNAs), and exon–intron circRNAs (EIciRNAs) (16, 43). EcircRNAs, which are composed exclusively of exonic sequences, represent the most abundant subtype and are predominantly localized in the cytoplasm. Functionally, they are best known for acting as competing endogenous RNAs that sequester microRNAs, thereby modulating post-transcriptional gene expression (44). In contrast, ciRNAs and EIciRNAs retain intronic sequences and are primarily enriched in the nucleus, where they participate in the regulation of transcription and alternative splicing. Mechanistically, these nuclear circRNAs can interact with components of the transcriptional machinery, such as U1 small nuclear ribonucleoproteins and RNA polymerase II, to enhance or fine-tune the expression of their parental genes (45). Importantly, current circRNA studies in sepsis-induced acute lung injury have largely focused on cytoplasmic ecircRNAs, particularly those involved in miRNA-mediated regulation of inflammation and cell death. The potential contributions of nuclear circRNAs to transcriptional reprogramming during septic lung injury, however, remain poorly understood and warrant further investigation. Recognizing this biogenetic and functional heterogeneity is essential for a more nuanced understanding of circRNA biology and for the rational design of circRNA-based diagnostic and therapeutic strategies.

In the context of sepsis-associated acute lung injury, circRNA biogenesis is likely influenced by the unique stress environment of the septic lung, including overwhelming inflammation, oxidative stress, and extensive transcriptional reprogramming. Dysregulation of splicing factors and RNA-binding proteins under septic conditions may selectively alter circRNA formation in alveolar epithelial cells, pulmonary endothelial cells, and lung-resident immune cells. Such context-dependent modulation of circRNA biogenesis may contribute to disease-specific circRNA expression patterns observed in sepsis-induced lung injury.

### 2.2 Modes of action

CircRNAs exert their biological functions through several distinct and often interconnected mechanisms, underscoring their versatility as regulators of gene expression (46, 47). One of the most widely studied functions is their role as competing endogenous RNAs (ceRNAs). CircRNAs frequently harbor multiple microRNA response elements (MREs), which enable them to act as molecular sponges that sequester microRNAs (miRNAs). By competitively binding miRNAs, circRNAs can relieve repression on downstream messenger RNAs (mRNAs), thereby modulating post-transcriptional gene expression (48, 49). This ceRNA activity has been implicated in numerous biological processes and pathological conditions, including inflammatory signaling, immune dysregulation, apoptosis, oxidative stress, and tumor progression (50, 51). In addition to miRNA sponging, circRNAs exert important functions through interactions with RNA-binding proteins (RBPs). They can serve as scaffolds, platforms, or decoys that influence the activity, stability, and subcellular localization of RBPs. By recruiting or sequestering RBPs, circRNAs are able to stabilize specific mRNAs, regulate protein translation, and modulate alternative splicing events. This expands their regulatory reach beyond miRNA networks and integrates them into broader RNA–protein interaction landscapes (52, 53). A subset of circRNAs, particularly those retained in the nucleus, also participates in transcriptional regulation. Nuclear circRNAs can modulate the expression of their parental genes by interacting with transcriptional machinery or chromatin-modifying complexes. For instance, certain exon–intron circRNAs (EIciRNAs) interact with U1 snRNP and RNA polymerase II, thereby promoting transcription of their host genes. This nuclear role highlights an additional level of gene regulatory capacity that distinguishes circRNAs from other ncRNA classes. Another intriguing function of circRNAs is their emerging ability to encode proteins or peptides (54, 55). Although traditionally considered non-coding, several circRNAs have been found to contain internal ribosome entry sites (IRES) or N6-methyladenosine (m6A) modifications that enable cap-independent translation. The peptides produced from circRNAs, although still incompletely characterized, may exert functional roles in signaling pathways, metabolism, or stress responses. This unexpected translational potential adds a new layer of complexity to circRNA biology and challenges the conventional distinction between coding and non-coding RNAs. Taken together, circRNAs operate through multiple complementary mechanisms—miRNA sequestration, RBP interactions, transcriptional regulation, and even translation—positioning them as multifunctional regulators within intricate molecular networks. These diverse modes of action not only broaden the biological impact of circRNAs but also highlight their potential relevance in complex diseases such as sepsis-induced acute lung injury.

### 2.3 Detection and functional validation

**Figure 1:**
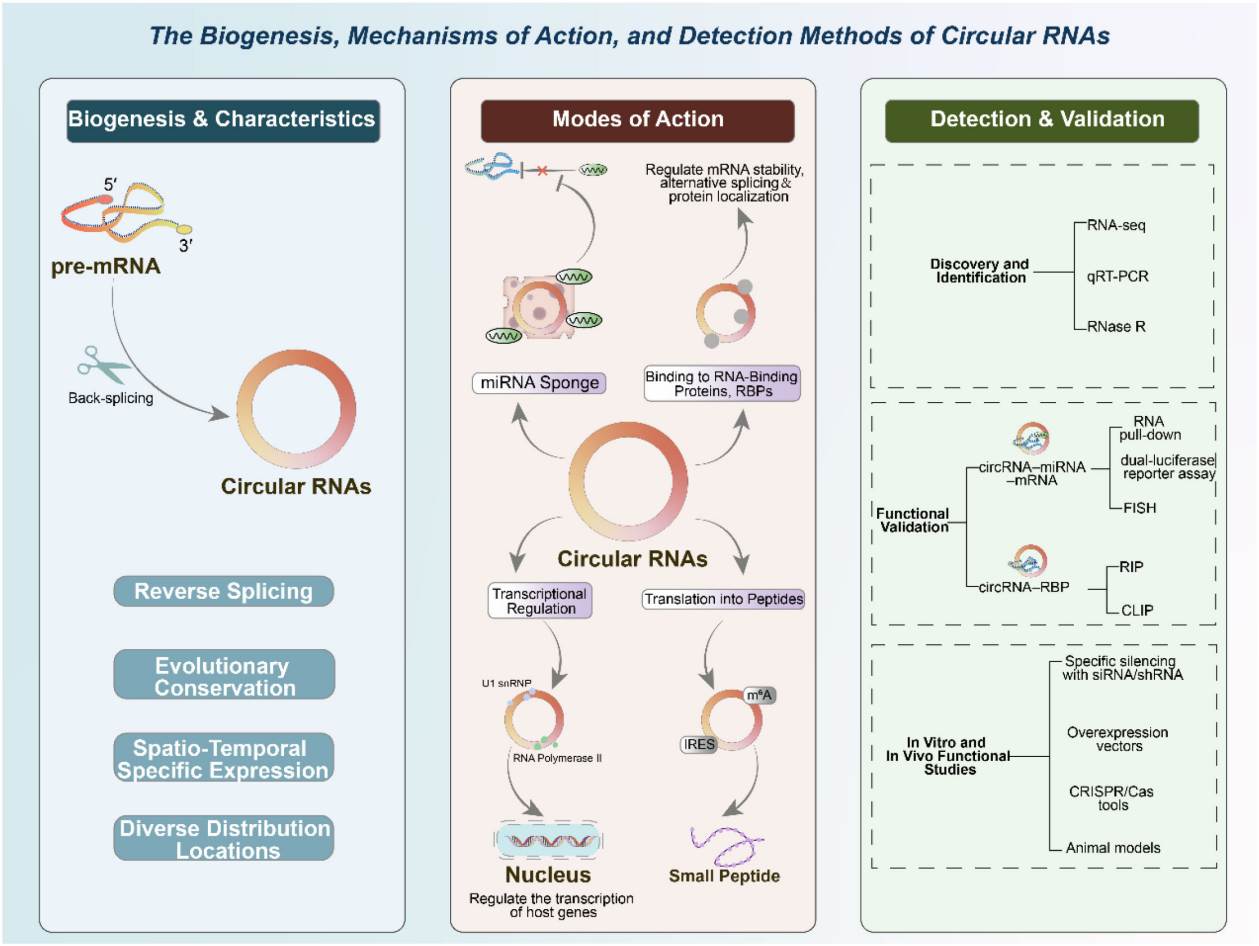
Biogenesis, modes of action, and detection methods of circRNAs.

The investigation of circRNAs has been greatly accelerated by the rapid development of high-throughput sequencing technologies and advanced molecular biology methods (56, 57). RNA sequencing (RNA-seq) remains the most powerful tool for circRNA discovery, as specialized bioinformatic pipelines can identify back-splice junction reads that uniquely distinguish circRNAs from their linear counterparts (58, 59). To ensure accuracy, computational prediction is often combined with experimental validation. Quantitative real-time PCR (qRT-PCR) using divergent primers spanning the circular junction has become the gold standard for confirming circRNA expression, while RNase R treatment is commonly applied to degrade linear RNAs and enrich circular forms, further validating their circular nature (60, 61). Beyond identification, a variety of functional assays are employed to characterize circRNA interactions and biological roles. For circRNA–miRNA–mRNA networks, dual-luciferase reporter assays, RNA pull-down experiments using biotin-labeled circRNA probes, and fluorescence *in situ* hybridization (FISH) are frequently used to verify direct molecular binding and spatial co-localization (62–64). For circRNA–protein interactions, RNA immunoprecipitation (RIP) and crosslinking immunoprecipitation (CLIP) approaches are used to capture circRNA–RBP complexes, enabling the identification of binding partners and downstream regulatory effects (65, 66). To assess functional relevance, loss-of-function and gain-of-function strategies are routinely applied. Small interfering RNAs (siRNAs) or short hairpin RNAs (shRNAs) designed to target the back-splice junction allow specific knockdown of circRNAs without affecting linear mRNA transcripts from the same host gene. Conversely, circRNA overexpression plasmids or lentiviral vectors can be used to elevate circRNA levels *in vitro* or *in vivo*. These approaches are complemented by CRISPR/Cas-based technologies, which are increasingly being developed for more precise manipulation of circRNA loci (67, 68). Importantly, animal models provide an indispensable platform for validating circRNA functions in disease contexts. By applying circRNA knockdown or overexpression strategies in models such as lipopolysaccharide (LPS)-induced acute lung injury or cecal ligation and puncture (CLP)-induced sepsis, researchers can evaluate the physiological relevance of candidate circRNAs in inflammation, immune responses, and lung barrier dysfunction. Moreover, the detection of circRNAs in circulating fluids such as plasma or bronchoalveolar lavage fluid (BALF) has facilitated translational studies, enabling the assessment of circRNAs as potential biomarkers for diagnosis and prognosis. Collectively, the integration of bioinformatic, molecular, and animal model approaches has firmly established circRNAs as key regulators of gene expression networks. These methodological advances not only provide mechanistic insights into circRNA biology but also lay the foundation for exploring their potential as diagnostic biomarkers and therapeutic targets in sepsis-induced acute lung injury and other critical illnesses. As shown in **Figure 1**, circRNAs arise from back-splicing of pre-mRNAs, forming stable covalently closed RNA circles that are conserved across species and exhibit specific expression patterns depending on tissue, developmental stage, and pathological context.

When circRNAs are evaluated as diagnostic or prognostic biomarkers, ensuring analytical specificity represents a critical technical challenge. Because circRNAs share extensive sequence overlap with their linear host transcripts, conventional RNA detection approaches may inadvertently capture linear RNAs, leading to false-positive signals (43, 69). To address this issue, circRNA detection typically relies on divergent primers specifically designed to span the back-splice junction, which is absent in linear RNAs. RNase R treatment is also widely used to selectively degrade linear RNAs and enrich circular transcripts, although incomplete digestion of certain structured linear RNAs should be carefully considered (17, 70). In translational settings, additional strategies such as junction-specific probes, paired quantification of corresponding linear transcripts, and digital droplet PCR have been proposed to further improve detection accuracy. Standardization of these approaches will be essential for the reliable clinical application of circRNA-based biomarkers.

## 3 CircRNAs in sepsis-induced acute lung injury: mechanistic insights

Accumulating evidence demonstrates that circRNAs participate in the regulation of multiple pathological processes in sepsis-induced acute lung injury (ALI). By functioning as molecular sponges for miRNAs, interacting with RNA-binding proteins, or modulating transcriptional and translational processes, circRNAs orchestrate key cellular events, including different forms of programmed cell death, immune cell activation, and barrier integrity (71, 72). Below, we summarize current findings by grouping circRNA functions into three major mechanistic categories.

### 3.1 Regulation of cell death pathways

**Figure 2:**
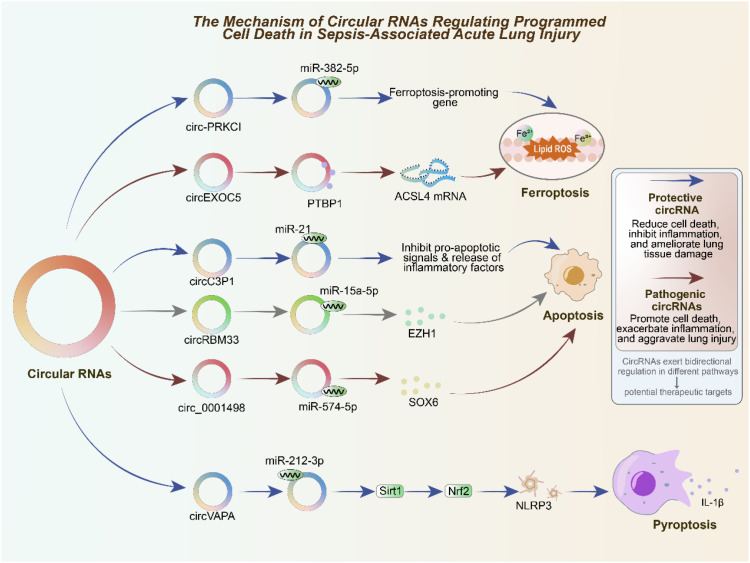
Mechanisms of circRNAs in regulating programmed cell death during sepsis-associated acute lung injury (ALI).

Programmed cell death represents a central pathological hallmark of sepsis-induced acute lung injury, particularly affecting alveolar epithelial cells, pulmonary endothelial cells, and lung-resident immune populations, and circRNAs have emerged as important modulators of multiple death pathways, including ferroptosis, apoptosis, and pyroptosis (14, 73). These regulated cell death processes are not isolated events but are intimately interconnected, collectively shaping the inflammatory microenvironment and determining the extent of lung tissue injury or repair during sepsis. Ferroptosis, an iron-dependent form of regulated necrosis characterized by excessive lipid peroxidation and membrane damage, has recently gained recognition as a key driver of alveolar epithelial cell injury in sepsis (74–76). Circ-PRKCI (hsa_circRNA_0122683) has been shown to play a protective role by sponging miR-382-5p, thereby preventing the depression of downstream pro-ferroptotic targets and ultimately reducing ferroptosis and inflammatory injury in pulmonary epithelial cells (30). In contrast, circEXOC5 functions as a pathogenic regulator by interacting with the RNA-binding protein PTBP1, which enhances the stability of ACSL4 mRNA, a central enzyme involved in lipid peroxidation. By amplifying ACSL4 expression, circEXOC5 aggravates ferroptotic damage, thereby worsening lung injury (77). Together, these findings highlight a dual regulatory paradigm in which circRNAs can either mitigate or exacerbate ferroptosis depending on their molecular partners. Apoptosis is another critical mechanism contributing to alveolar and endothelial dysfunction in septic lungs (78). Circ_0001498 promotes apoptosis by sequestering miR-574-5p and upregulating SOX6, thereby driving epithelial cell death and amplifying inflammatory responses (72). Conversely, circC3P1 exerts a protective role through direct interaction with miR-21, leading to suppression of pro-apoptotic signaling, attenuation of inflammatory cytokine release, and reduced endothelial cell apoptosis (79). CircRBM33 adds further complexity by regulating apoptosis via competitive binding to miR-15a-5p, which in turn controls the expression of EZH1, a key epigenetic regulator (80). This suggests that circRNAs can influence apoptosis not only by directly modulating death-related genes but also by engaging with broader chromatin-remodeling pathways that affect cell survival and inflammation. Pyroptosis, a highly inflammatory form of programmed cell death triggered by inflammasome activation and caspase-1-mediated cleavage of gasdermins, is increasingly recognized as a pivotal contributor to cytokine release and tissue injury in sepsis (81, 82). CircVAPA has been identified as an anti-pyroptotic regulator, functioning by sponging miR-212-3p, thereby relieving repression of Sirt1. Activated Sirt1 subsequently promotes Nrf2 signaling and suppresses NLRP3 inflammasome activation, ultimately attenuating macrophage pyroptosis and reducing lung inflammation (36, 83). Collectively, these studies demonstrate that circRNAs are versatile regulators of programmed cell death in sepsis-induced ALI, acting at the crossroads of ferroptosis, apoptosis, and pyroptosis. Importantly, their effects are context-dependent: certain circRNAs act as protective molecules that limit tissue injury, while others serve as pathogenic drivers that amplify inflammatory damage. Understanding the interplay between these circRNA-mediated pathways may provide novel opportunities for therapeutic intervention, particularly by targeting key circRNA–miRNA–mRNA regulatory axes to rebalance cell death and survival in the injured lung. As illustrated in **Figure 2**, circRNAs exert critical roles in regulating programmed cell death pathways in sepsis-associated ALI.

### 3.2 Regulation of immune cells and inflammatory signaling

**Figure 3:**
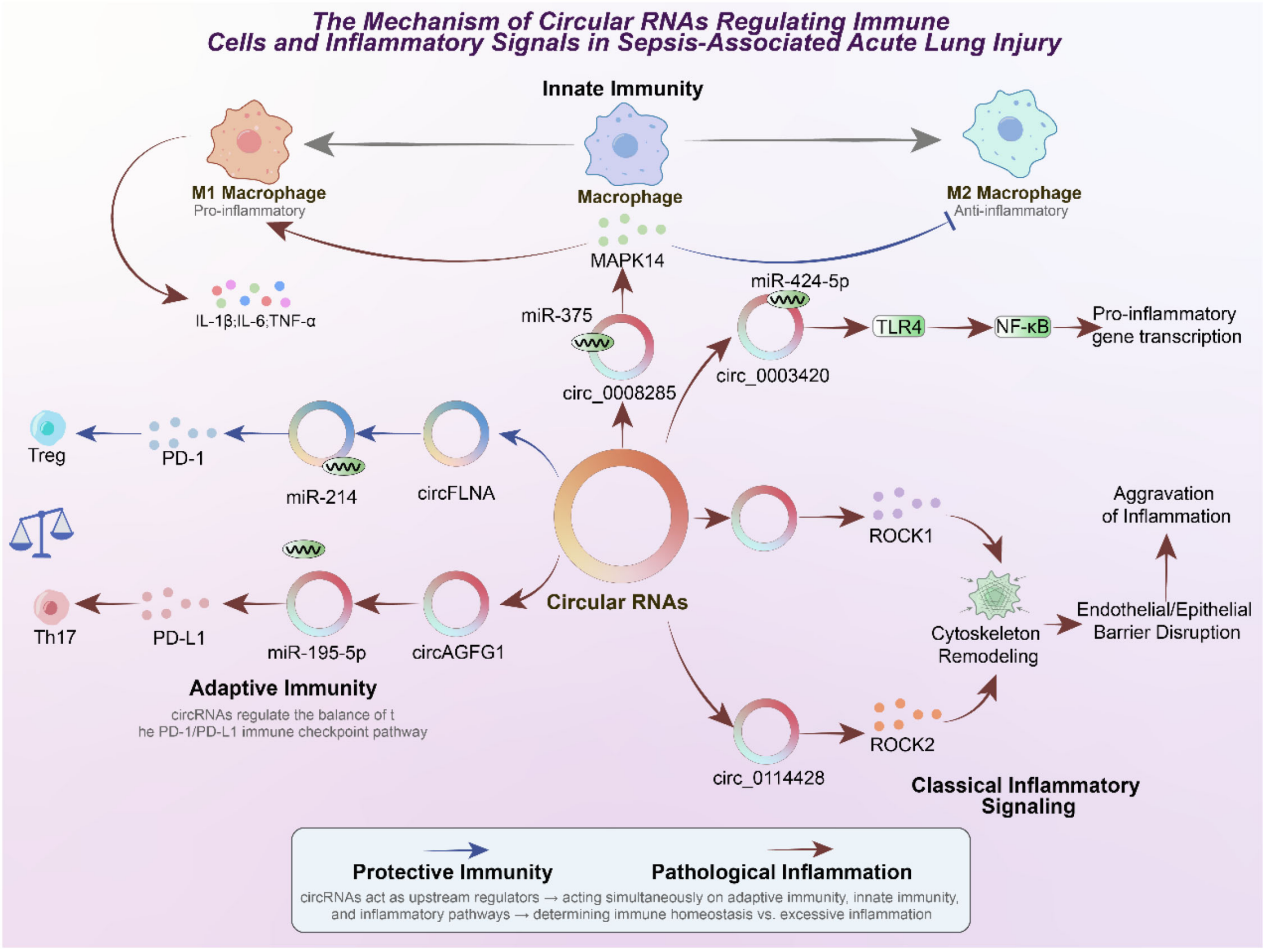
Regulatory mechanisms of circRNAs in immune cell function and inflammatory signaling during sepsis-associated acute lung injury (ALI).

In addition to orchestrating cell death pathways, circRNAs play critical roles in shaping immune responses and modulating inflammatory signaling cascades, thereby influencing both the initiation and resolution of sepsis-induced acute lung injury (ALI) (84, 85). In sepsis-associated lung injury, these circRNA-mediated immune regulatory mechanisms are especially relevant within the pulmonary microenvironment, where dysregulated macrophage activation, T cell imbalance, and cytokine amplification directly drive tissue damage and disease progression. Dysregulated immunity is a hallmark of sepsis: excessive activation of pro-inflammatory pathways drives tissue injury, while compensatory immunosuppression impairs host defense (86). CircRNAs have been shown to act at the interface of adaptive and innate immunity, exerting regulatory effects on T cell differentiation, macrophage polarization, and classical inflammatory signaling pathways (87–89). The balance between regulatory T cells (Tregs) and T helper 17 (Th17) cells is crucial for maintaining immune homeostasis in sepsis. Tregs suppress excessive inflammation, while Th17 cells promote neutrophil recruitment and tissue injury. CircRNAs have been implicated in tipping this balance. For instance, circFLNA promotes PD-1 expression by sponging miR-214, thereby enhancing Treg differentiation and immunosuppressive function (90). In parallel, circAGFG1 acts via the miR-195-5p/PD-L1 axis to influence immune checkpoint pathways and modulate Th17 responses (91). These findings highlight the involvement of circRNAs in the regulation of adaptive immunity, linking them to immune checkpoint signaling—a mechanism typically studied in oncology but increasingly relevant in septic immunopathology. Beyond adaptive immunity, circRNAs shape the activity of macrophages, which act as central mediators of lung inflammation. Circ_0008285, for example, drives macrophage polarization toward the pro-inflammatory M1 phenotype by sponging miR-375 and upregulating MAPK14 (p38 MAPK). This shift enhances the production of inflammatory cytokines such as IL-1β, IL-6, and TNF-α while suppressing the anti-inflammatory M2 phenotype, thereby exacerbating tissue damage (71). This illustrates how circRNAs can dictate innate immune cell fate and function, ultimately influencing the inflammatory milieu of the septic lung. In addition to cell-specific effects, circRNAs regulate canonical signaling cascades that underpin inflammatory responses. Circ_0003420 enhances TLR4 expression by sequestering miR-424-5p, leading to NF-κB activation and amplification of pro-inflammatory gene transcription (92). Similarly, circKLHL2 and circ_0114428 modulate ROCK1 and ROCK2 via interactions with miR-29b-3p and miR-574-5p, respectively (93, 94). By influencing Rho/ROCK signaling, these circRNAs contribute to cytoskeletal remodeling, disruption of epithelial and endothelial barriers, and heightened inflammatory injury. Such findings reinforce the concept that circRNAs function as upstream molecular switches that fine-tune multiple interconnected inflammatory pathways. Collectively, these studies establish circRNAs as pivotal regulators of immune and inflammatory dynamics in sepsis-induced ALI. By modulating both adaptive and innate immune responses, as well as key signaling cascades, circRNAs dictate the balance between protective immune regulation and pathological hyperinflammation. Importantly, their ability to regulate immune checkpoints and macrophage polarization positions them at the crossroads of immune homeostasis and dysregulation, suggesting that circRNAs may serve not only as mechanistic drivers of sepsis pathology but also as attractive targets for immunomodulatory therapy. As illustrated in **Figure 3**, circRNAs function as critical regulators of immune responses in sepsis-associated ALI. Within adaptive immunity, circFLNA promotes Treg differentiation through PD-1 upregulation, thereby maintaining immune suppression, whereas circAGFG1 enhances PD-L1 expression and favors Th17-driven inflammatory responses.

### 3.3 Endothelial and epithelial barrier protection

**Figure 4:**
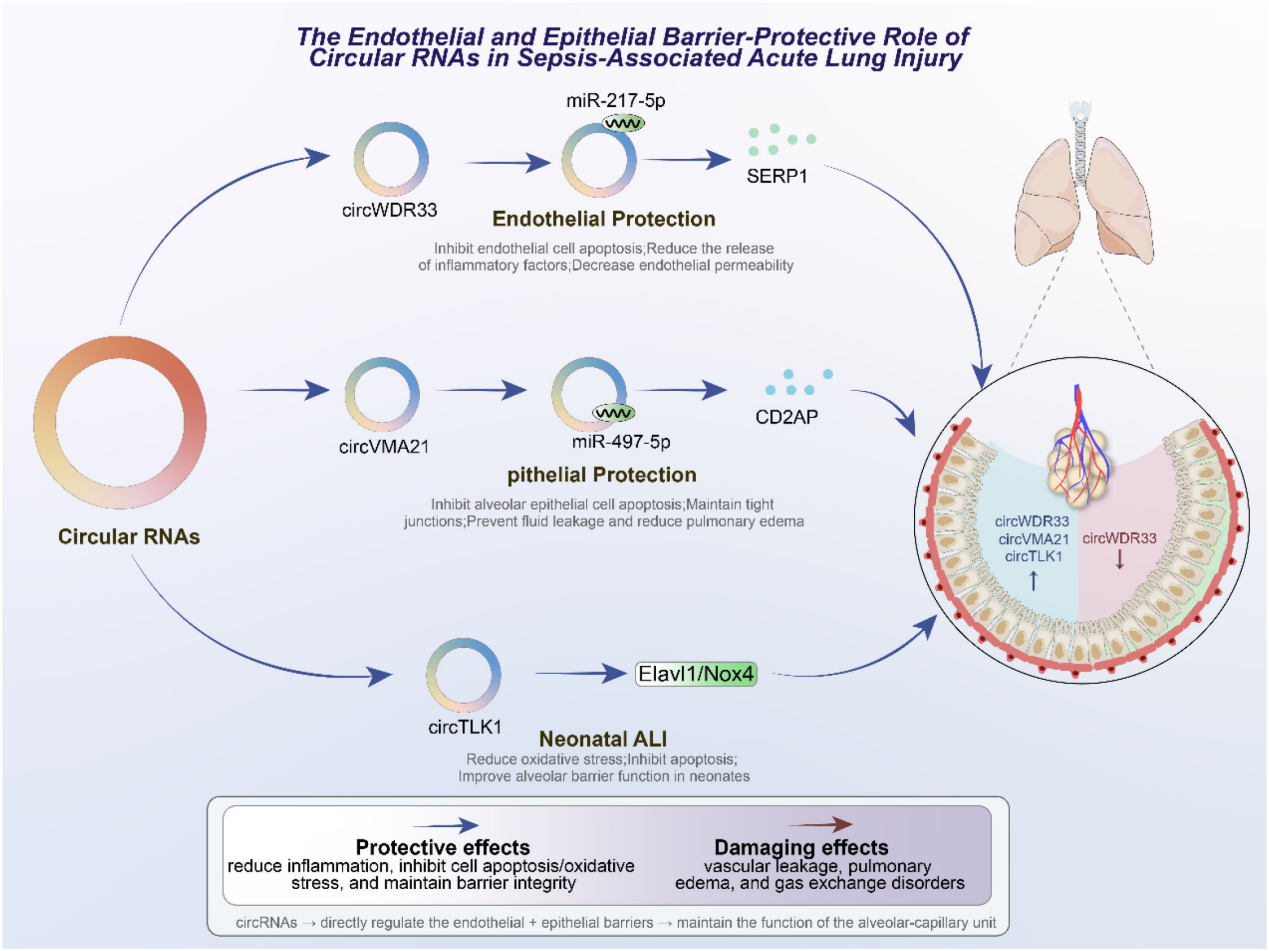
Protective roles of circRNAs in endothelial and epithelial barrier integrity during sepsis-associated acute lung injury (ALI).

Given the central role of the alveolar–capillary unit in gas exchange, circRNA-mediated regulation of endothelial and epithelial barrier integrity represents a lung-specific mechanism that critically shapes the severity of sepsis-associated acute lung injury. Disruption of pulmonary barrier integrity is a defining feature of sepsis-induced acute lung injury (ALI), characterized by increased alveolar-capillary permeability, pulmonary edema, and impaired gas exchange. Both endothelial cells, which line the pulmonary microvasculature, and alveolar epithelial cells, which form the interface with the external environment (8, 95), play indispensable roles in maintaining lung barrier function. CircRNAs have emerged as important modulators of barrier stability, acting through diverse molecular pathways to counteract inflammatory injury, oxidative stress, and apoptotic signaling (28, 96). Endothelial cell dysfunction contributes directly to vascular leakage and inflammatory cell infiltration during sepsis. CircWDR33 has been shown to exert a protective role in pulmonary microvascular endothelial cells by sponging miR-217-5p, thereby upregulating SERP1, a protein involved in endoplasmic reticulum stress regulation. This circRNA-mediated pathway reduces inflammatory cytokine release, inhibits apoptosis, and decreases endothelial permeability, ultimately preserving vascular integrity (97). Such findings suggest that targeting circWDR33 or its downstream effectors may represent a promising strategy to restore endothelial homeostasis in septic ALI. The alveolar epithelium is equally critical for barrier function, preventing fluid leakage into the alveolar space and maintaining efficient gas exchange. CircVMA21 has been identified as a protective circRNA in this context, acting through the miR-497-5p/CD2AP axis to attenuate epithelial cell death and preserve tight junction integrity (98). By sustaining epithelial barrier function, circVMA21 limits pulmonary edema and reduces tissue damage, highlighting the importance of circRNAs in epithelial resilience under septic conditions. Interestingly, circRNAs also appear to regulate barrier function in age-dependent contexts. In neonatal models of sepsis-induced ALI, circTLK1 has been shown to modulate oxidative stress and apoptosis via the Elavl1/Nox4 pathway. By reducing oxidative injury and apoptotic signaling, circTLK1 knockdown alleviates barrier dysfunction in neonatal lungs (99). This suggests that circRNA-mediated protective mechanisms are not restricted to adult models but are conserved across developmental stages, broadening their clinical relevance. Collectively, these studies demonstrate that circRNAs contribute directly to the maintenance of pulmonary barrier integrity, complementing their roles in immune regulation and cell death pathways. By targeting endothelial and epithelial cells, circRNAs safeguard structural and functional aspects of the alveolar-capillary unit, which is central to lung physiology and pathology in sepsis. These findings not only underscore the mechanistic diversity of circRNA actions but also provide strong rationale for their exploration as therapeutic targets aimed at preserving or restoring barrier function in septic patients. As illustrated in **Figure 4**, circRNAs exert barrier-protective functions in sepsis-associated ALI by targeting both endothelial and epithelial compartments of the alveolar–capillary unit.

## 4 Translational perspectives

### 4.1 Biomarker potential

One of the most promising aspects of circRNAs lies in their potential as diagnostic and prognostic biomarkers for sepsis-induced ALI. Unlike linear RNAs, circRNAs possess a covalently closed circular structure that makes them inherently resistant to exonuclease-mediated degradation (100–102). This unique feature confers exceptional stability, allowing circRNAs to accumulate and persist in diverse biological samples, including peripheral blood, bronchoalveolar lavage fluid (BALF), urine, and extracellular vesicles such as exosomes. Their stability in circulation, even under harsh physiological conditions, provides a significant advantage over other classes of non-coding RNAs for use in clinical testing. In addition to their structural resilience, circRNAs exhibit tissue-specific, cell-type-specific, and even disease-stage-specific expression patterns. Such specificity suggests that circRNA signatures could serve as sensitive indicators of pathological changes in the septic lung (31, 98, 103). Importantly, circRNAs can be readily detected in a minimally invasive manner, making them suitable for liquid biopsy approaches. For example, transcriptome-wide sequencing efforts, such as the study by Xu et al. (2024), have identified panels of circRNAs differentially expressed in sepsis-associated ALI, thereby providing valuable candidate molecules for biomarker development (100). Some circRNAs may correlate with disease severity, while others could serve as predictors of treatment response or long-term outcomes, thus enabling stratification of patients according to risk and prognosis. Moreover, circRNAs packaged into exosomes hold additional translational value. Exosomal circRNAs are not only highly stable but also reflect the physiological state of their cells of origin, offering a dynamic snapshot of disease progression (85, 104). Given that exosomes can cross biological barriers, exosomal circRNAs may also participate in intercellular communication during sepsis, linking their role as biomarkers to potential mechanistic significance (105, 106). Incorporating circRNA-based assays into clinical practice could therefore enhance the precision of early diagnosis, disease monitoring, and prognostic evaluation in septic patients. When integrated with established clinical scoring systems and conventional biomarkers such as procalcitonin or C-reactive protein, circRNA signatures may significantly improve the accuracy of patient stratification and decision-making in intensive care. However, large-scale, multicenter validation studies will be essential to confirm their diagnostic power and establish standardized protocols for circRNA detection and quantification.

### 4.2 Therapeutic potential

Beyond their value as biomarkers, circRNAs also represent attractive therapeutic targets in sepsis-induced acute lung injury (ALI). Preclinical studies have provided proof-of-concept evidence that modulation of circRNA expression can significantly influence disease outcomes (72, 100, 101). Silencing pathogenic circRNAs using small interfering RNAs (siRNAs) or short hairpin RNAs (shRNAs) specifically targeting back-splice junctions has been shown to reduce inflammation, limit apoptosis, attenuate oxidative stress, and restore barrier function in septic lung models (92, 98, 99). Conversely, overexpression of protective circRNAs through plasmid or viral vector-based systems can enhance cellular resilience, suppress excessive immune activation, and promote tissue repair. These findings support the feasibility of circRNA-targeted interventions as a novel therapeutic strategy in sepsis. Recent advances in drug delivery systems have further expanded the translational potential of circRNA-based therapies (107–109). Traditional approaches, such as naked siRNAs, often suffer from poor stability, off-target effects, and low delivery efficiency. To overcome these barriers, researchers are increasingly leveraging nanoparticle-based platforms (e.g., lipid nanoparticles, polymeric nanoparticles) to encapsulate circRNA modulators, thereby improving stability and enabling targeted delivery to the lung (110, 111). Similarly, exosome-mediated delivery systems—which exploit the natural transport function of extracellular vesicles—offer an innovative means of achieving high biocompatibility, reduced immunogenicity, and tissue-specific targeting. These delivery technologies are particularly promising in the context of sepsis, where systemic inflammation and vascular leakage complicate drug distribution. Despite these advances, several challenges remain. CircRNAs that regulate immune checkpoint pathways (e.g., PD-1/PD-L1 axes) illustrate the complexity of therapeutic applications. While inhibiting such circRNAs may alleviate lung inflammation and tissue injury, unchecked suppression of immune checkpoints could also impair host defense, increasing susceptibility to secondary infections. This “double-edged sword” effect underscores the importance of achieving precise temporal and spatial control over circRNA-targeted interventions (31, 65, 112). Furthermore, off-target effects, long-term safety, and the risk of unintended modulation of host immune homeostasis remain significant concerns that require systematic evaluation in large-animal models before clinical translation. In summary, circRNAs offer a promising therapeutic avenue for sepsis-induced ALI by targeting central mechanisms of inflammation, cell death, and barrier dysfunction. The integration of circRNA modulation with emerging delivery technologies has the potential to transform current treatment paradigms. However, careful optimization of delivery systems, dosing strategies, and safety profiles will be essential to balance efficacy with host immune competence in the septic setting.

### 4.3 Challenges and limitations

**Figure 5:**
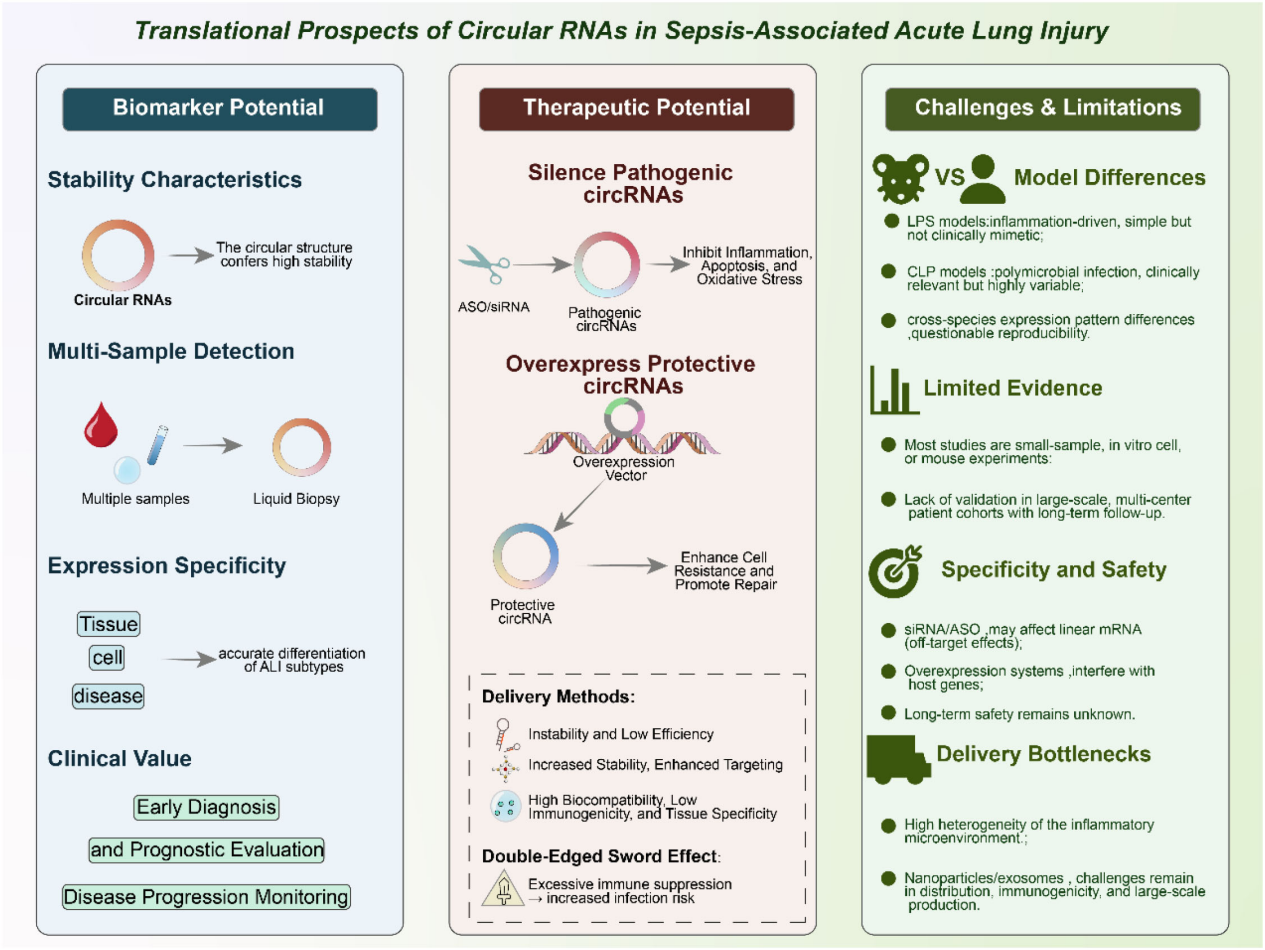
Translational potential of circRNAs in sepsis-associated acute lung injury (ALI).

Despite the encouraging preclinical evidence, several challenges continue to hinder the clinical translation of circRNA research in sepsis-induced acute lung injury (ALI). First, discrepancies among experimental models represent a major obstacle. Lipopolysaccharide (LPS)-induced models are widely used due to their simplicity and reproducibility, but they primarily capture endotoxin-driven inflammation and may not fully replicate the complexity of human sepsis (113, 114). Cecal ligation and puncture (CLP), considered a more clinically relevant model, induces polymicrobial infection and systemic inflammation but is technically variable and associated with high mortality. Patient-derived clinical samples add another layer of complexity, as circRNA expression patterns can differ substantially from those observed in animal models, raising concerns about reproducibility, translatability, and the generalizability of preclinical findings (115–117). Second, the strength of the current evidence base remains limited. Most existing studies rely on small-scale preclinical experiments involving cell lines or rodent models, often with modest sample sizes. While these studies provide valuable mechanistic insights, they lack the statistical power and external validation required for clinical application. To date, evidence supporting circRNA function in sepsis-induced ALI is largely confined to expression profiling and experimental manipulation in preclinical systems, whereas interventional clinical trials and robust functional validation in human tissues remain lacking. Large, multicenter patient cohorts with longitudinal follow-up will therefore be essential to confirm the diagnostic, prognostic, and therapeutic potential of circRNAs in sepsis-induced ALI. Without such validation, the risk of overinterpreting preclinical findings remains substantial. CircRNA knockdown strategies using siRNAs or antisense oligonucleotides (ASOs) face the risk of off-target effects due to sequence overlap with linear mRNA isoforms, complicating the interpretation of functional outcomes. Similarly, overexpression systems may unintentionally alter the expression of host genes, creating artifacts that obscure the true contribution of circRNAs (118, 119). In addition, the long-term safety of circRNA modulation is largely unknown, as perturbing circRNA–miRNA–mRNA regulatory networks may disrupt fundamental cellular processes beyond the intended targets, particularly in the context of systemic inflammation. Finally, delivery remains a major translational bottleneck. Effective therapeutic modulation of circRNAs requires delivery systems capable of achieving cell-type specificity within the inflamed and highly heterogeneous microenvironment of the septic lung. Current approaches using lipid nanoparticles, polymeric carriers, or exosome-based systems are promising but continue to face challenges related to biodistribution, immune activation, large-scale manufacturing, and regulatory oversight. In particular, ethical and logistical considerations surrounding exosome sourcing and standardization further complicate their clinical applicability. Moreover, emerging high-resolution approaches such as single-cell and spatial transcriptomics, while powerful for mechanistic discovery, are associated with high costs and limited accessibility, restricting their widespread use in large sepsis cohorts and real-world clinical settings. Importantly, therapeutic targeting of circRNAs involved in immune regulatory pathways may also carry potential risks. Given the well-recognized phenomenon of sepsis-induced immune paralysis, interventions affecting immune checkpoint–related axes, although beneficial in oncology, could inadvertently exacerbate immunosuppression in sepsis, underscoring the need for cautious, context-dependent evaluation. In light of these challenges, future progress will depend on the development of standardized experimental methodologies, the establishment of large-scale patient validation studies, and the refinement of innovative delivery technologies tailored for the pulmonary system. Only by systematically addressing these limitations can circRNA-based strategies move from proof-of-concept studies toward tangible diagnostic and therapeutic applications in sepsis-induced ALI. **Figure 5** illustrates the translational landscape of circRNAs in sepsis-induced ALI, highlighting both opportunities and limitations. Representative circRNAs implicated in sepsis-induced ALI, together with their proposed mechanisms and levels of supporting evidence, are summarized in **Table 1**.

**Table 1:** Representative circRNAs involved in sepsis-induced acute lung injury.

circRNA	Proposed mechanism	Functional role in ALI	Evidence level	References
circPRKCI (hsa_circRNA_0122683)	miRNA sponging (miR-382-5p)	Attenuates ferroptosis and inflammatory injury	Preclinical	(30)
circEXOC5	PTBP1–ACSL4 stabilization	Aggravates ferroptotic lung injury	Preclinical	(77)
circ_0001498	miR-574-5p/SOX6 axis	Promotes epithelial apoptosis	Preclinical	(72)
circC3P1	miR-21-mediated signaling	Protects endothelial cells from apoptosis	Preclinical	(101)
circRBM33	miR-15a-5p/EZH1 axis	Epigenetic regulation of apoptosis	Preclinical	(80)
circVAPA	miR-212-3p/Sirt1/Nrf2 axis	Suppresses macrophage pyroptosis	Preclinical	(36, 83)
circFLNA	miR-214/PD-1 axis	Promotes Treg differentiation	Preclinical	(90)
circAGFG1	miR-195-5p/PD-L1 axis	Regulates Th17 responses	Preclinical	(91)
circ_0008285	miR-375/MAPK14 axis	Drives M1 macrophage polarization	Preclinical	(71)
circ_0003420	miR-424-5p/TLR4/NF-κB axis	Amplifies inflammatory signaling	Preclinical	(92)
circWDR33	miR-217-5p/SERP1 axis	Preserves endothelial barrier integrity	Preclinical	(97)
circVMA21	miR-497-5p/CD2AP axis	Protects epithelial barrier function	Preclinical	(98)
circTLK1	Elavl1/Nox4 signaling	Modulates oxidative stress in neonatal ALI	Preclinical	(99)

## 5 Conclusion

CircRNAs have rapidly emerged as critical regulators in the pathogenesis of sepsis-induced acute lung injury (ALI), demonstrating both pathogenic and protective functions depending on their molecular targets, signaling partners, and cellular context. On the one hand, several circRNAs act as drivers of lung injury, promoting inflammatory cascades, facilitating apoptosis or ferroptosis, and destabilizing epithelial and endothelial barriers. On the other hand, an equally important group of circRNAs function as protective mediators, capable of suppressing pyroptosis, attenuating apoptosis, maintaining barrier integrity, and fine-tuning immune responses. This duality underscores the context-dependent nature of circRNA regulation and highlights their role as key modulators of the delicate balance between injury and repair in septic lungs. Mechanistically, circRNAs orchestrate a wide range of processes that are central to sepsis-induced ALI, including multiple forms of programmed cell death (apoptosis, ferroptosis, pyroptosis), immune cell regulation (Treg/Th17 balance, macrophage polarization, and checkpoint signaling), and the preservation of alveolar-capillary barrier function (94, 120, 121). By integrating these diverse pathways, circRNAs appear to operate as central hubs within complex gene regulatory networks, amplifying or dampening pathological signals in a cell- and pathway-specific manner. Such integrative functions make circRNAs particularly attractive as both mechanistic entry points and therapeutic intervention targets. Looking ahead, the unique stability, abundance, and tissue-specific expression patterns of circRNAs underscore their strong potential as diagnostic and prognostic biomarkers. Their detectability in blood and exosomes also offers practical opportunities for minimally invasive monitoring, with the potential to complement or even surpass existing biomarkers in sensitivity and specificity. Furthermore, the growing body of preclinical evidence suggests that circRNAs may be therapeutically actionable, whether through silencing pathogenic species or restoring protective ones. Nevertheless, several critical challenges remain before circRNAs can be translated into clinical practice. These include discrepancies between animal models and human disease, the lack of large multicenter patient cohorts for validation, technical issues related to detection and quantification, and unresolved questions about the specificity, safety, and efficiency of circRNA-targeted therapies. Overcoming these barriers will require coordinated efforts that combine rigorous mechanistic research, advanced delivery technologies, and standardized clinical protocols. In conclusion, circRNAs represent a promising frontier in sepsis research, bridging basic mechanistic insights with translational opportunities. By continuing to unravel their multifaceted roles in programmed cell death, immune regulation, and barrier function, future studies may pave the way for circRNA-based strategies to improve the diagnosis, prognosis, and treatment of sepsis-induced ALI. If successfully translated, circRNA-targeted approaches have the potential to transform current paradigms in critical care medicine and offer new hope for patients facing this devastating condition.
